# Physiological, Cytological, and Transcriptomic Analysis of Magnesium Protoporphyrin IX Methyltransferase Mutant Reveal Complex Genetic Regulatory Network Linking Chlorophyll Synthesis and Chloroplast Development in Rice

**DOI:** 10.3390/plants12213785

**Published:** 2023-11-06

**Authors:** Youming Yao, Hongyu Zhang, Rong Guo, Jiangmin Fan, Siyi Liu, Jianglin Liao, Yingjin Huang, Zhaohai Wang

**Affiliations:** 1 Key Laboratory of Crop Physiology, Ecology and Genetic Breeding (Jiangxi Agricultural University), Ministry of Education of the P.R. China, Nanchang 330045, China; youming_yao@163.com (Y.Y.); jn_zhanghongyu@163.com (H.Z.); rong_guo1582@163.com (R.G.); jiangminfzz@163.com (J.F.); lsy64112138@163.com (S.L.); jlliao514815@163.com (J.L.); yjhuang_cn@126.com (Y.H.); 2 Key Laboratory of Agriculture Responding to Climate Change (Jiangxi Agricultural University), Nanchang 330045, China

**Keywords:** magnesium protoporphyrin IX methyltransferase (ChlM), chlorophyll synthesis, chloroplast development, hormone, rice

## Abstract

Functional defects in key genes for chlorophyll synthesis usually cause abnormal chloroplast development, but the genetic regulatory network for these key genes in regulating chloroplast development is still unclear. Magnesium protoporphyrin IX methyltransferase (ChlM) is a key rate-limiting enzyme in the process of chlorophyll synthesis. Physiological analysis showed that the chlorophyll and carotenoid contents were significantly decreased in the *chlm* mutant. Transmission electron microscopy demonstrated that the chloroplasts of the *chlm* mutant were not well developed, with poor, loose, and indistinct thylakoid membranes. Hormone content analysis found that jasmonic acid, salicylic acid, and auxin accumulated in the mutant. A comparative transcriptome profiling identified 1534 differentially expressed genes (DEGs) between *chlm* and the wild type, including 876 up-regulated genes and 658 down-regulated genes. Gene Ontology (GO) and Kyoto Encyclopedia of Genes and Genomes (KEGG) analysis revealed that these DEGs were highly involved in chlorophyll metabolism, chloroplast development, and photosynthesis. Protein−protein interaction network analysis found that protein translation played an essential role in the *ChlM* gene-regulated process. Specifically, 62 and 6 DEGs were annotated to regulate chlorophyll and carotenoid metabolism, respectively; 278 DEGs were predicted to be involved in regulating chloroplast development; 59 DEGs were found to regulate hormone regulatory pathways; 192 DEGs were annotated to regulate signal pathways; and 49 DEGs were putatively identified as transcription factors. Dozens of these genes have been well studied and reported to play essential roles in chlorophyll accumulation or chloroplast development, providing direct evidence for the reliability of the role of the identified DEGs. These findings suggest that chlorophyll synthesis and chloroplast development are actively regulated by the *ChlM* gene. And it is suggested that hormones, signal pathways, and transcription regulation were all involved in these regulation processes. The accuracy of transcriptome data was validated by quantitative real-time PCR (qRT-PCR) analysis. This study reveals a complex genetic regulatory network of the *ChlM* gene regulating chlorophyll synthesis and chloroplast development. The *ChlM* gene’s role in retrograde signaling was discussed. Jasmonic acid, salicylic acid, or their derivatives in a certain unknown state were proposed as retrograde signaling molecules in one of the signaling pathways from the chloroplast to nucleus.

## 1. Introduction

Chlorophyll is widely found in photosynthetic organisms such as green plants, cyanobacteria, and algae [1]. It plays a central role in photosynthesis, forming complexes with thylakoid membrane proteins such as photosystem I, photosystem II, and cytochrome b6f complexes, which are important for regulating the photosynthetic physiology, yield, and quality in crops [2,3]. The biosynthesis of chlorophyll in higher plants is a complex process involving multiple enzymes, starting with glutamyl-tRNA and ending with the synthesis of chlorophyll *b*. This process consists of 16 steps, with more than 20 genes encoding 16 enzymes involved in the biosynthesis of chlorophyll [4].

In rice, 11 genes involved in chlorophyll biosynthesis have been cloned. *RLIN1* (LOC_Os04g52130, *HEMF1*) encodes a coproporphyrinogen oxidative decarboxylase, which converts coproporphyrinogen III to protoporphyrinogen IX in the chlorophyll biosynthesis pathway, and the mutation of the RLIN1 gene causes a small distortion in the thylakoid in the chloroplast structure [5,6]. *ChlD* (LOC_Os03g59640), *ChlH* (LOC_Os03g20700), and *ChlI* (LOC_Os03g36540) are predicted to encode the D, H, and I subunits of Mg-chelatase, respectively, which are involved in the synthesis of Mg-protoporphyrin IX from protoporphyrin IX in the chlorophyll biosynthesis pathway. Mutants of these three genes all showed decreased chlorophyll contents and disrupted the thylakoid membranes of the chloroplast [7,8]. *CRD1* (LOC_Os01g17170) encodes a monomethyl ester cyclase of magnesium protoporphyrin IX, converting Mg protoporphyrin IX monomethyl ester to form divinyl protochlorophyllide, and the mutation of this gene leads to chlorophyll synthesis deficiency and a lack of stacked grana thylakoids in the chloroplast [9]. *PORA* (LOC_Os04g58200) and *PORB* (LOC_Os10g35370) encode NADPH-protochlorophyllide oxidoreductases A and B, catalyzing the synthesis of chlorophyllide a from protochlorophyllide. *OsPORB* is required for the maintenance of light-dependent chlorophyll synthesis throughout leaf development, especially under high light levels, whereas *OsPORA* acts primarily in the early stages of leaf development; the *OsPORB* gene mutant showed severe leaf chlorosis accompanied by barely detectable thylakoid stacking and significantly increased plastoglobuli in the chloroplast [10]. *ChlG* (LOC_Os05g28200) encodes chlorophyll synthase, catalyzing the synthesis of chlorophyll *a* or *b* from chlorophyllide a or chlorophyllide *b*. The *ChlG* mutation results in a severe decline in chlorophyll and lacked grana membrane in the chloroplast [11]. Both *CAO1/PGL* (LOC_Os10g41780) and *CAO2* (LOC_Os10g41760) encode chlorophyllide *a* oxygenase 1, converting chlorophyllide *a* into chlorophyllide *b* in the chlorophyll synthesis pathway, and the *OsCAO1* mutants show a reduced chlorophyll content and disordered grana thylakoid of chloroplast [12,13]. It is clear from these studies that functional defects of key genes in chlorophyll synthesis also usually cause abnormal chloroplast development, but the downstream genetic networks for these key genes regulating chloroplast development are still unclear.

Magnesium protoporphyrin IX methyltransferase (ChlM) is a key rate-limiting enzyme in the process of chlorophyll synthesis. ChlM is synthesized in the cytoplasm and then transported to the chloroplast, where it is localized to the chloroplast envelope and thylakoid membrane, catalyzing the methyl transfer of S-adenosyl methionine to magnesium protoporphyrin IX (MgP) to form S-adenosyl homocysteine and magnesium protoporphyrin IX methyl ester (MgPME) in the chlorophyll synthesis pathway [14]. Two *chlamydomonas reinhardtii* mutants defective in the *ChlM* gene accumulated MgP and showed light-sensitive chlorophyll deficiency, and many photosynthesis-related chloroplast protein-encoding genes were regulated, such as *LHCB*, *ChlH*, *ChlI*, *ChlD*, *CRD1,* and *HEMA* [15]. In tobacco, ChlM has been shown to interact with the ChlH subunit of magnesium-chelatase to function and regulate the expression of chlorophyll-synthesis-related genes, such as *HEMA*, *GSA,* and *ChlH* [16]. Mutation in the *ChlM* gene in *Arabidopsis* resulted in the *chlm* mutant showing yellow–white leaves and accumulated MgP, and it also greatly changed the expression levels of some important chloroplast protein-encoding genes like *LHCB*, *RBCS*, and *ChlH* [17]. In addition, we previously cloned the rice *ChlM* gene using a yellow–green leaf mutant *ygll8*, and in vitro enzyme activity assays demonstrated that the YGL18 protein exhibited ChlM enzyme activity, but the mutant ygl18 protein retained only very little ChlM activity [18]. Accordingly, the substrate MgP accumulated in large amounts in ygl18 leaves, while the product MgPME greatly reduced. YGL18 protein is required for light-dependent and photoperiod-regulated chlorophyll synthesis [18]. Taken together, mutants of the *ChlM* gene in various plant species exhibited severe chlorophyll deficiency and significant expression alteration of many important chloroplast protein-encoding genes. The *chlm* mutant might be an ideal material to study the genetic regulation network critically linking chlorophyll synthesis and chloroplast development.

In this study, we analyzed the changes in pigment content, chloroplast structure, and hormone content in the rice *chlm* mutant and then used transcriptomic data to reveal the gene expression network of the *ChlM* gene regulating chlorophyll synthesis and chloroplast development. Genes related to pigment metabolism, chloroplast development, hormone pathways, signal pathways, and transcriptional regulation were found to play important roles in this regulation process. This study reveals a complex genetic regulatory network linking chlorophyll synthesis and chloroplast development in rice.

## 2. Results

### 2.1. Alteration of Pigment Contents and Chloroplast Structure in the Chlm Mutant

**Figure 1:**
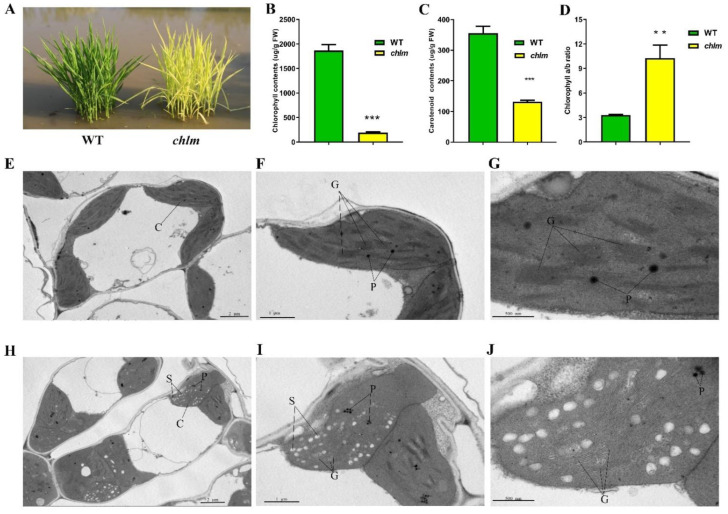
Phenotypic, physiological and cytological changes between wild-type (WT) and *chlm* mutant at the seedling stage. (**A**) Phenotype of wild-type and *chlm* mutant plants. (**B**) The chlorophyll contents of wild-type and *chlm* mutant leaves. (**C**) The carotenoid contents of wild-type and *chlm* mutant leaves. (**D**) The ratio of chlorophyll *a* to chlorophyll *b*. In (**B**–**D**), all data represent the mean ± SD of three biological replicates, and the asterisk indicates the statistically significant difference between *chlm* and wild type (** *p* < 0.005, *** *p* < 0.0005, Student’s *t*-test). (**E**–**G**) Chloroplast ultrastructure showing typical structures and distinct thylakoid membranes in wild-type leaves. (**H**–**J**) Abnormal chloroplast ultrastructure in the *chlm* mutant leaves. Bars = 2 μm (**E**,**H**), 1 μm (**F**,**I**), 500 nm (**G**,**J**). C, chloroplast. P, plastoglobuli. S, starch granule. G, grana stacks.

The *ChlM* gene mutant *chlm* was isolated from a photo-thermosensitive genic male sterile rice cultivar ‘guangzhan63S’ (*Oryza sativa* L. subsp. indica) by our previous study [18]. At the seedling stage, the mutant *chlm* could be easily distinguished by the yellow–green leaf trait (Figure 1A). Thus, the physiological changes of the *chlm* mutant were investigated. Compared with that of the wild type, the chlorophyll and carotenoid contents in the *chlm* mutant were significantly reduced (Figure 1B,C), and the ratio of chlorophyll *a* to chlorophyll *b* was significantly increased (Figure 1D), which was possibly due to the chlorophyll *b* synthesis suffering a more severe suppression than chlorophyll *a*. These results all indicated the impairment of pigment synthesis in the *chlm* mutant.

To determine whether the *ChlM* gene mutation affected chloroplast development, chloroplast structure changes of the *chlm* mutant leaf were additionally observed at the cytological level by using a transmission electron microscope. In the leaves of the wild type, the chloroplast showed the typical structure of well-developed and distinct thylakoid membranes with normal stacked grana (Figure 1E–G). In contrast, ultrastructural analysis of chloroplasts in the *chlm* mutant leaves revealed poor, loose and indistinct thylakoid membranes and increased osmiophilic plastoglobuli (Figure 1H–J). These results indicated that chloroplast development was significantly affected in the *chlm* mutant.

### 2.2. Hormone Changes in the Chlm Mutant

**Figure 2:**
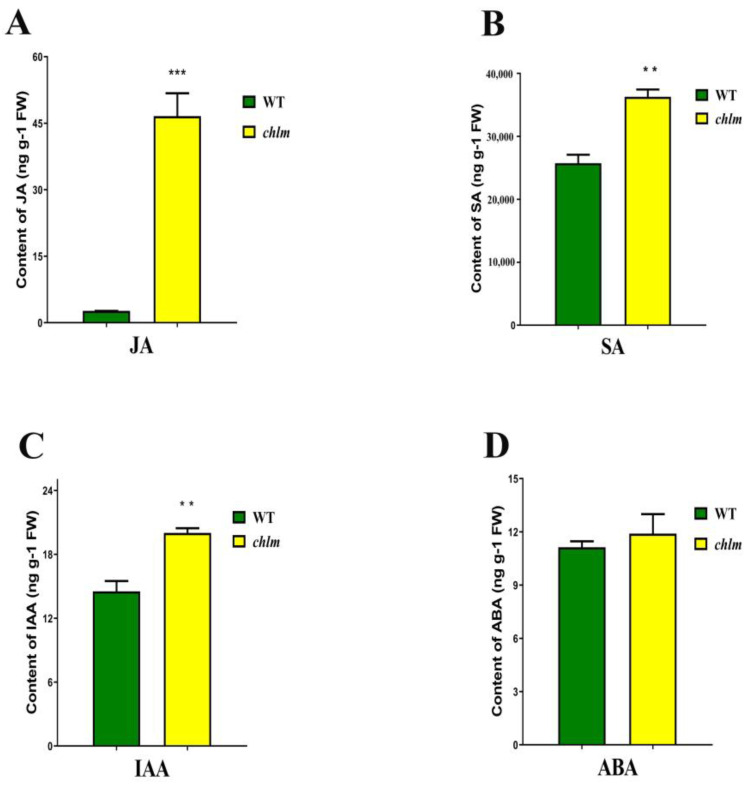
Changes of hormone contents between wild-type and *chlm* mutant leaves at the seedling stage. (**A**) The content of jasmonic acid (JA). (**B**) The content of salicylic acid (SA). (**C**) The content of indoleacetic acid (IAA). (**D**) The content of abscisic acid (ABA). All data represent the mean ± SD of three biological replicates, and the asterisk indicates the statistically significant difference between *chlm* and the wild type (** *p* < 0.005, *** *p* < 0.0005, Student’s *t*-test).

In parallel with the evident chloroplast structure changes in the *chlm* mutant, the contents of four phytohormones related with chloroplast were measured, including jasmonic acid (JA), salicylic acid (SA), indole acetic acid (IAA) and abscisic acid (ABA). Compared with that of the wild type, the contents of JA, SA and IAA were significantly increased in the *chlm* mutant, especially the highest increase for JA (Figure 2A–C). Meanwhile, the content of ABA showed no difference between the wild type and the *chlm* mutant (Figure 2D). The increased JA, SA and IAA may function in the biological process regulated by the *ChlM* gene.

### 2.3. Transcriptomic Alterations in Chlm Mutant

Considering the significant changes of phenotypic, physiological and cytological characteristics of the *chlm* mutant in this study, it was worth investigating the transcriptional regulation network of the *ChlM* gene. Thus, a transcriptomic comparison between the yellow–green leaves of the *chlm* mutant with the normal green leaves of the wild type was carried out by RNA sequencing of six libraries with three biological replicates each. A total of 40.10 Gb clean reads (accounting for 92.19% of the raw reads) were obtained by stringent quality check and data cleanup, and the clean reads of each sample were more than 6 Gb. In detail, the total clean reads in each library mapped to the *Oryza sativa* reference genome ranged 86.72–87.15%, and the unique reads mapped to the rice reference genome ranged 82.61–83.93% (Table 1). In addition, the GC contents of the sequencing outputs were 54.56–55.14%, and the Q30 percentage of clean reads was greater than 85% (Table 1). Accordingly, the throughput and quality of RNA sequencing were high enough to ensure further analysis.

### 2.4. Analysis of Differentially Expressed Genes (DEGs)

**Figure 3:**
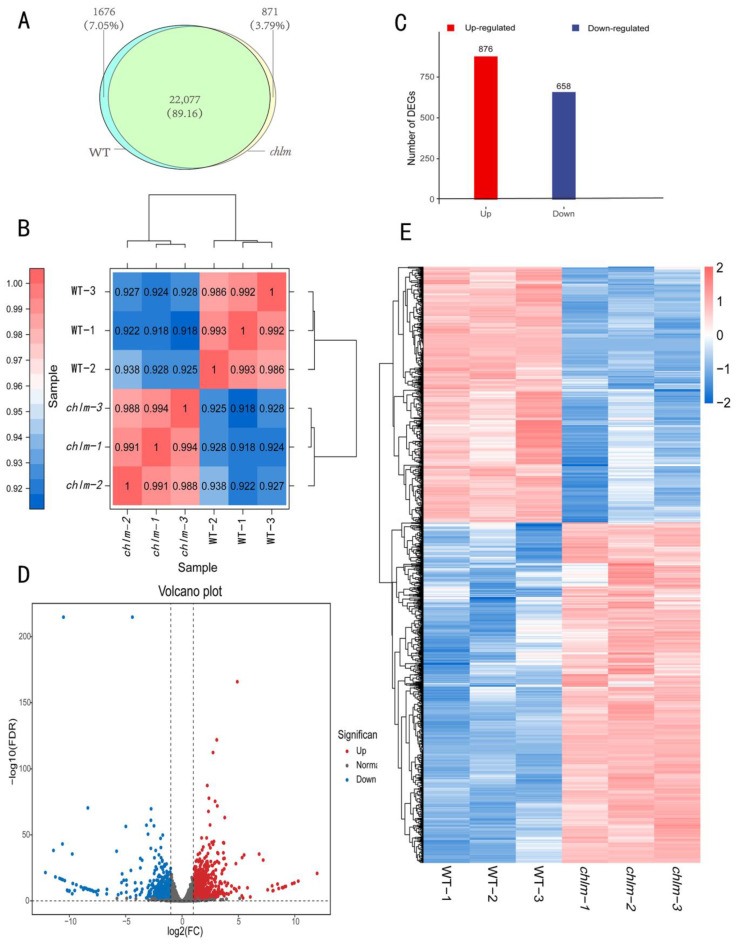
The identification and analysis of differentially expressed genes (DEGs). (**A**) Number of genes identified in wild type (WT) and the *chlm* mutant. (**B**) The correlation of biological replicates evaluated by Pearson’s correlation coefficient. (**C**) The number of up- and down-regulated genes. (**D**) The volcano plot of DEGs. (**E**) The hierarchical clustering of DEGs.

After clean reads were mapped to the Oryza sativa reference genome, the mapped transcripts were assembled and annotated using StringTie software (1.3.4d). Furthermore, FPKM (Fragments Per Kilobase of transcript per Million fragments mapped) methods were used to analyze gene expression patterns in both types of libraries. When comparing the two types of libraries with respect to the FPKM calculation, 23,753 (wild-type) and 22,948 (*chlm*) genes were identified in the cDNA libraries; meanwhile, 22,077 genes were simultaneously expressed in wild-type and *chlm* mutant leaves (Figure 3A). Prior to identifying DEGs, the correlation of biological replicates was evaluated. The result displayed a great repeatability (Figure 3B) and thus could be provided for further analysis. According to the screening criteria of false discovery rate (FDR) < 0.01 and fold change ≥ 2, a total of 1534 DEGs were obtained between *chlm* and the wild type (Table S1). Among them, 876 genes were up-expressed, while 658 genes were down-expressed (Figure 3C). In addition, the volcano plot and hierarchical clustering analysis of DEGs directly showed the differences of gene expression level between the *chlm* mutant and the wild type (Figure 3D,E).

### 2.5. Functional Analysis of DEGs

**Figure 4:**
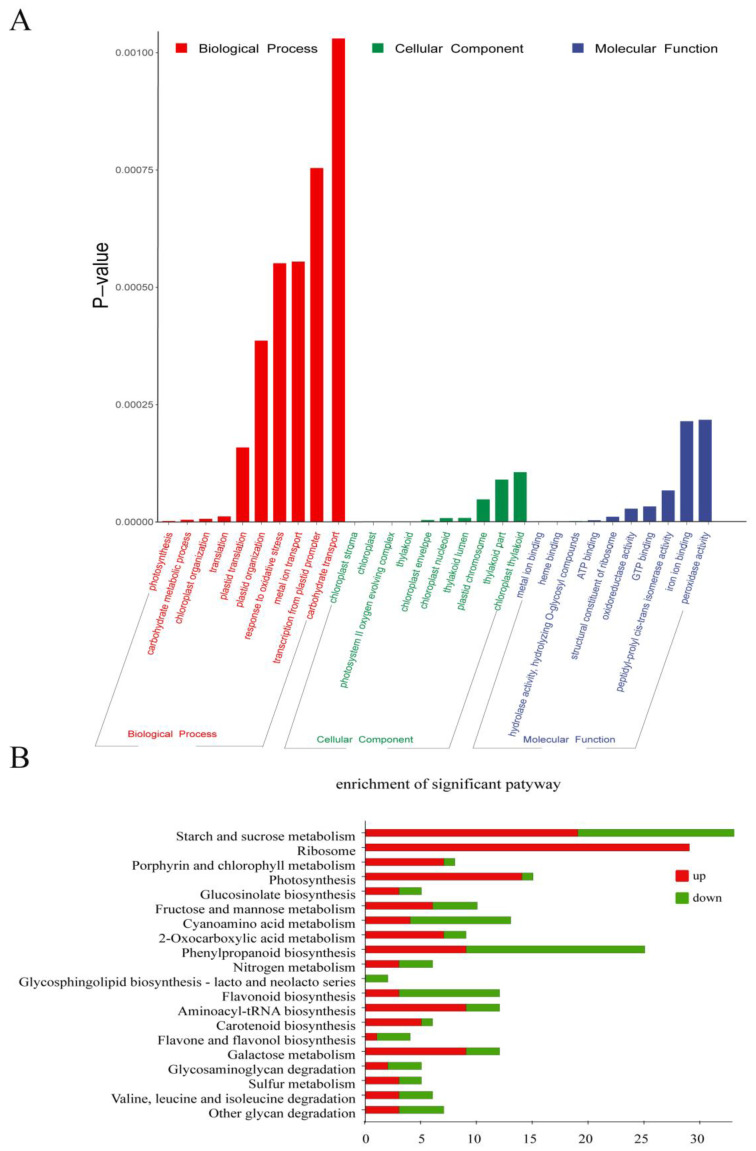
Functional enrichment analysis of DEGs between the wild type (WT) and the *chlm* mutant. (**A**) Gene Ontology (GO) enrichment analysis of DEGs. The 30 most enriched GO terms in three categories are shown. (**B**) Kyoto Encyclopedia of Genes and Genomes (KEGG) analysis of DEGs. The top 20 most significant pathways are shown.

To investigate the potential functions of DEGs involved in the biological process regulated by the *ChlM* gene, functional enrichment analysis on 1534 DEGs was performed. Gene Ontology (GO) enrichment analysis results showed that a total of 257 GO terms were significantly enriched (*p*-value < 0.05), among which 112, 39 and 106 were classified under biological process, cellular component, and molecular function, respectively (Table S2). Moreover, a total of 30 significantly top terms were additionally listed (Figure 4A). In biological process terms, ‘photosynthesis (GO:0015979)’, ‘carbohydrate metabolic process (GO:0005975)’, ‘chloroplast organization (GO:0009658)’, ‘translation (GO:0006412)’, ‘plastid translation (GO:0032544)’, ‘plastid organization (GO:0009657)’, ‘response to oxidative stress (GO:0006979)’, ‘metal ion transport (GO:0030001)’, ‘transcription from plastid promoter (GO:0042793)’, and ‘carbohydrate transport (GO:0008643)’ were significantly enriched. In cellular component terms, DEGs were mainly enriched in ‘chloroplast stroma (GO:0009570)’, ‘chloroplast (GO:0009507)’, ‘photosystem II oxygen evolving complex (GO:0009654)’, ‘thylakoid (GO:0009579)’, ‘chloroplast envelope (GO:0009941)’, ‘chloroplast nucleoid (GO:0042644)’, ‘thylakoid lumen (GO:0031977)’, ‘plastid chromosome (GO:0009508)’, ‘thylakoid part (GO:0044436)’, and ‘chloroplast thylakoid (GO:0009534)’, which met the expectation. And in molecular function terms, DEGs were significantly enriched in ‘metal ion binding (GO:0046872)’, ‘heme binding (GO:0020037)’, ‘hydrolase activity, hydrolyzing O-glycosyl compounds (GO:0004553)’, ‘ATP binding (GO:0005524)’, ‘structural constituent of ribosome (GO:0003735)’, ‘oxidoreductase activity (GO:0016491)’, ‘GTP binding (GO:0005525)’, ‘peptidyl-prolyl cis-trans isomerase activity (GO:0003755)’, ‘iron ion binding (GO:0005506)’, and ‘peroxidase activity (GO:0004601)’. Obviously, these GO terms are highly related to chlorophyll metabolism and chloroplast development.

Furthermore, Kyoto Encyclopedia of Genes and Genomes (KEGG) analysis was performed to further explore the metabolic pathways of DEGs involved in a *ChlM*-regulated biological process. At last, a total of 20 pathways were significantly enriched with a *p*-value < 0.05, in which ‘starch and sucrose metabolism (ko00500)’, ‘ribosome (ko03010)’, ‘porphyrin and chlorophyll metabolism (ko00860)’, ‘photosynthesis (ko00195)’, ‘glucosinolate biosynthesis (ko00966)’, and ‘fructose and mannose metabolism (ko00051)’ were the top six pathways (Figure 4B, Table S2). Apparently, these pathways are also highly related with chlorophyll metabolism and photosynthesis.

### 2.6. Functional Interaction Network of DEGs

**Figure 5:**
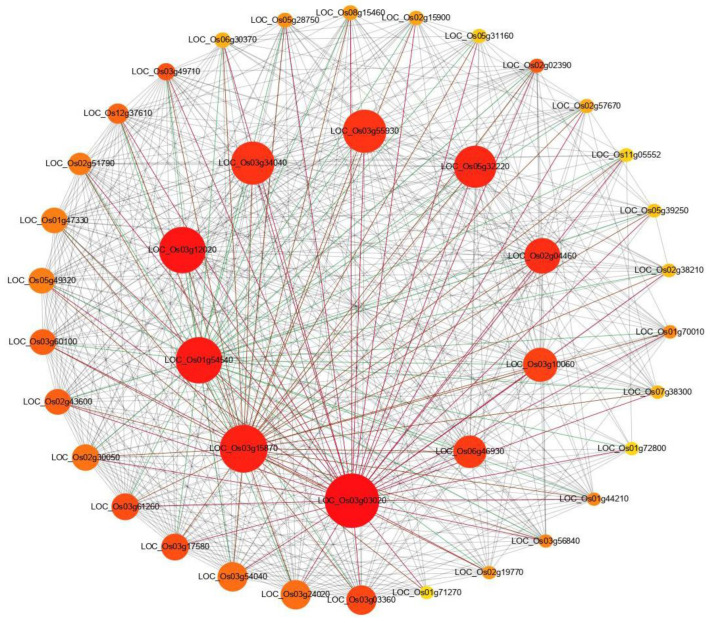
Protein–protein interaction network analysis of DEGs. STRING V9.1 software was used to predict the protein to protein network of differentially expressed proteins. Differentially accumulated proteins are represented by a node, whereas the different color of lines represents evidence for the predicted functional relationship. The strong interaction is indicated by a redder color. The proteins outside the circle showed weaker interaction.

To reveal the functional interaction network of DEGs, the protein–protein interaction (PPI) analysis was conducted using all identified DEGs between the wild type and the *chlm* mutant. Of the predicted PPI network, highly connected nodes are central to a network’s architecture and function. The top 40 most significant proteins with the highest connectivity were extracted from the predicted network. The result showed that higher interactions were found for proteins encoded by LOC_Os03g03020 (50S ribosomal protein L11), LOC_Os03g15870 (50S ribosomal protein L4), LOC_Os01g54540 (50S ribosomal protein L13), LOC_Os03g12020 (50S ribosomal protein L15), LOC_Os03g34040 (30S ribosomal protein S5), LOC_Os03g55930 (30S ribosomal protein S9), LOC_Os05g32220 (50S ribosomal protein L1), LOC_Os02g04460 (50S ribosomal protein L3), LOC_Os03g10060 (30S ribosomal protein S10), and LOC_Os06g46930 (50S ribosomal protein L24) (Figure 5, Table S3), all being ribosomal proteins for translation. The remaining 30 proteins were also related with ribosome or translation (Table S3). And 39 of these 40 protein-encoding genes were up-regulated in the *chlm* mutant (Table S3). The above results implied that the translation process played an essential role in chlorophyll synthesis and chloroplast development regulated by the *ChlM* gene.

### 2.7. Genes Regulating Chlorophyll and Carotenoid Metabolism Were Identified in the Chlm Mutant

**Figure 6:**
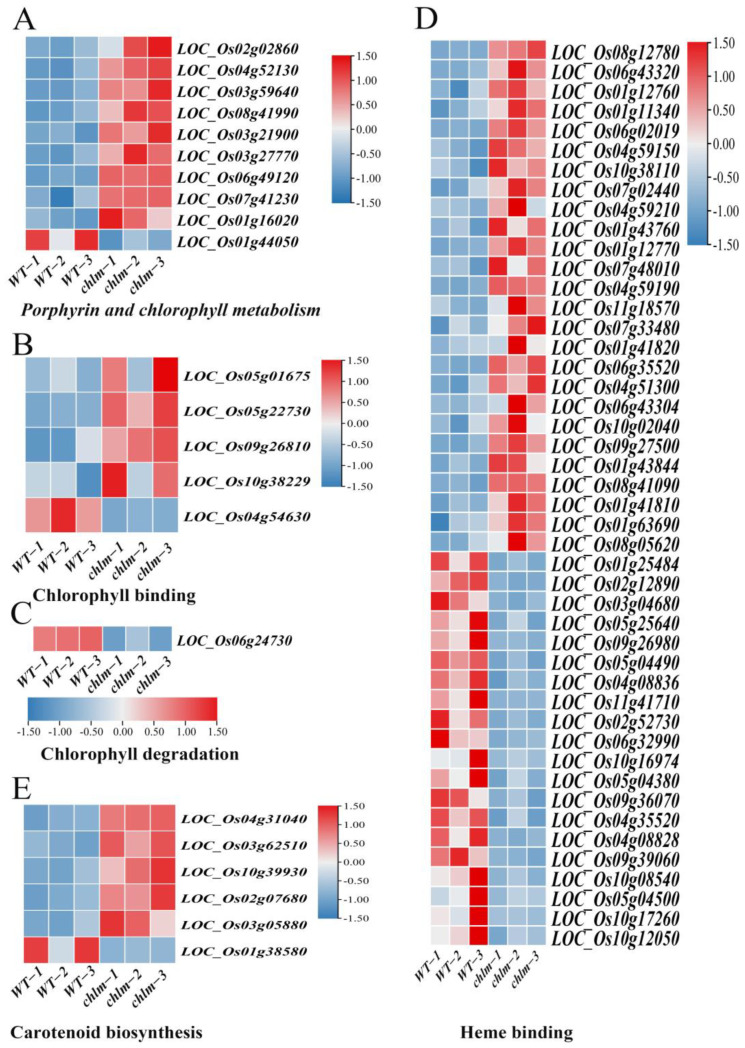
Expression patterns of DEGs regulating chlorophyll and carotenoid metabolism pathways in the *chlm* mutant. (**A**) DEGs involved in porphyrin and chlorophyll metabolism. (**B**) DEGs involved in chlorophyll binding. (**C**) DEGs involved in chlorophyll degradation. (**D**) DEGs involved in heme binding. (**E**) DEGs involved in carotenoid biosynthesis.

Chlorophyll is the main component of the photosynthetic pigments, which performs important functions in photosynthesis by harvesting light energy and converting it into chemical energy. Chlorophyll synthesis and heme synthesis are two branches in the tetrapyrrole biosynthesis pathway, Mg chelating with protoporphyrin IX to step into the chlorophyll synthesis branch and Fe chelating with protoporphyrin IX to step into the heme synthesis branch [1]. In this study, a total of 62 chlorophyll-related DEGs were identified based on functional annotation, including 39 up-regulated genes and 23 down-regulated genes. These DEGs were assigned to relevant functional terms: specifically, 10 genes involved in porphyrin and chlorophyll metabolism (Figure 6A, Table S4), five genes involved in chlorophyll binding (Figure 6B, Table S4), one gene involved in chlorophyll degradation (Figure 6C, Table S4), and 46 genes involved in heme binding (Figure 6D, Table S4). Among these DEGs, some were cloned and well-studied. The up-expressed genes LOC_Os02g02860 (*OsGluRS*) encoding a glutamyl tRNA synthetase, LOC_Os04g52130 (*RLIN1*, *LLM1*) encoding a putative coproporphyrinogen III oxidase, and LOC_ Os03g59640 (*OsChlD*) encoding the ChlD subunit of Mg-protoporphyrin IX chelatase were well-studied key enzymes for chlorophyll biosynthesis (Table 2). The down-expressed gene LOC_Os06g24730 (*NYC3*) encoding an α/β fold hydrolase family protein played an important role in chlorophyll degradation (Table 2). And the up-expressed gene LOC_Os03g27770 (*OsHO2*) encoding the heme oxygenase was reported to play a key role in heme biosynthesis (Table 2). These results suggested that the chlorophyll metabolism pathway is regulated by the *ChlM* gene.

Furthermore, carotenoids are a diverse group of colorful pigments naturally found in plants, which play essential roles in photosynthesis and plant development [53]. In this study, a total of six carotenoid biosynthesis-related DEGs were identified based on functional annotation, including five up-regulated genes and one down-regulated gene (Figure 6E, Table S4). These results implied that the carotenoid biosynthesis pathway is regulated by the *ChlM* gene.

These results implied that the information for the decreased content of chlorophyll and carotenoids in the *chlm* mutant may be perceived by plant cells, which further regulate the expression level of these pigments’ related biosynthetic and metabolic genes, trying to compensate for the reduction in chlorophyll and carotenoids due to the impaired function of the *ChlM* gene in the *chlm* mutant.

### 2.8. Genes Regulating Chloroplast Development Were Identified in the Chlm Mutant

Chloroplasts are the central nodes of the metabolic network in the photosynthetic cells of higher plants, which performs indispensable functions in photosynthesis and other metabolic processes, such as the synthesis of lipid, terpenoids, tetrapyrroles, amino acids and hormones [54]. Many gene mutants in the chlorophyll synthesis pathway have showed defects in chloroplast structure or development. Since the *chlm* mutant also showed weakened chloroplast development except for the decreased chlorophyll contents, it would be interesting to investigate the expression profile of genes related with chloroplast.

**Figure 7:**
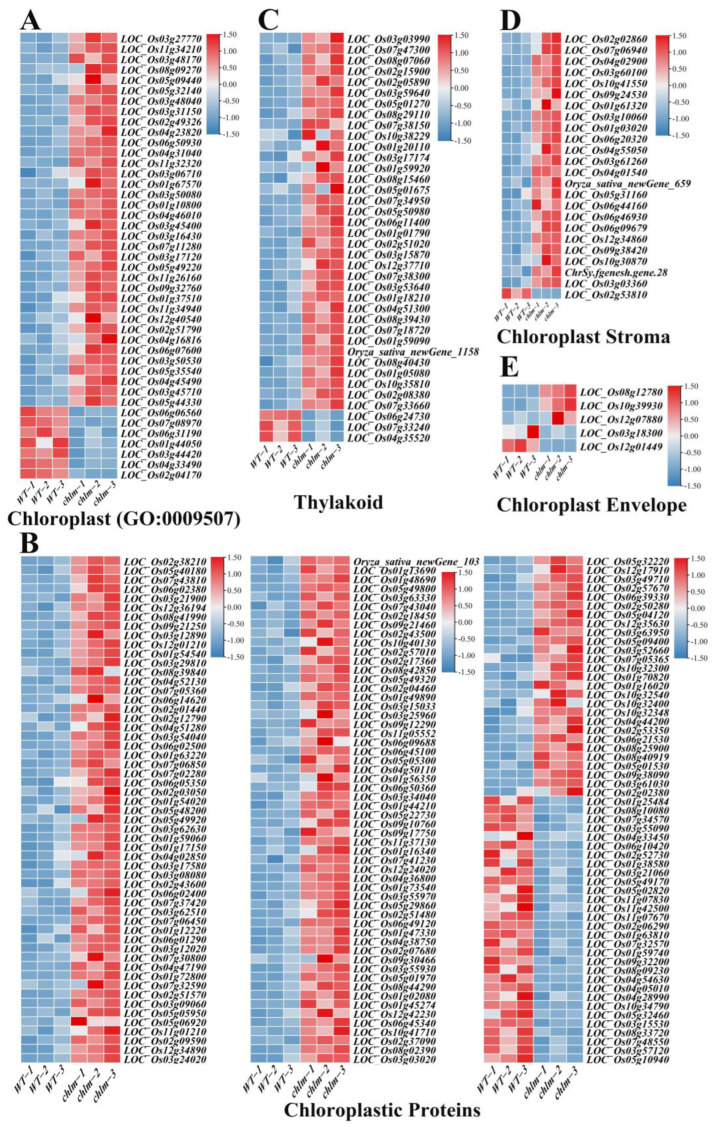
Expression patterns of DEGs regulating chloroplast development in the *chlm* mutant. (**A**) DEGs referring to cellular component chloroplast (GO:0009507). (**B**) DEGs referring to chloroplastic proteins. (**C**) DEGs related with thylakoid. (**D**) DEGs related with chloroplast stroma. (**E**) DEGs related with chloroplast envelope.

In this study, 43 genes were functionally annotated to be related with cellular component chloroplast (GO:0009507) (Figure 7A, Table S5). And 168 genes were functionally annotated to encode chloroplastic proteins (Figure 7B, Table S5). Specifically, 38, 24 and 5 genes were functionally annotated to be related to the thylakoid, chloroplast stroma, and chloroplast envelope, respectively (Figure 7C–E, Table S5). Obviously, of these 278 chloroplast-related genes, the vast majority (235) were up-regulated in the *chlm* mutant (Table S5), suggesting the positive response of these genes in chloroplast development. Through searching these 278 chloroplast-related genes on ‘China Rice Data Center’, 26 well-studied genes were reported to be involved in chlorophyll metabolism and chloroplast development (Table 2), including *WSP1* (LOC_Os04g51280), *OsTrxZ/wp2* (LOC_Os08g29110), *WLP2* (LOC_Os01g63220), *OsPPR6* (LOC_Os05g49920), *CDE4* (LOC_Os08g09270), *etl1* (LOC_Os11g01210), *etl2* (LOC_Os12g01210), *WSL3* (LOC_Os10g32540), *OsNUS1* (LOC_Os03g45400), *WLP1* (LOC_Os01g54540), *ASL2* (LOC_Os02g15900), *OsValRS2* (LOC_Os07g06940), *ObgC* (LOC_Os07g47300), *EF-Tu* (LOC_Os02g38210), *YL1* (LOC_Os02g05890), *OscpSRP43* (LOC_Os03g03990), *YGL138(t)* (LOC_Os11g05552), *AL1* (LOC_Os03g31150), *OsNOA1* (LOC_Os02g01440), *VYL* (LOC_Os03g29810), *OsFdC1* (LOC_Os03g45710), *OsFdC2* (LOC_Os03g48040), *OsSTN8* (LOC_Os05g40180), *RNRS1* (LOC_Os06g14620), *YLC1* (LOC_Os09g21250), and *WSL12* (LOC_Os12g36194) (Table 2). These well-studied genes provided direct evidence for the reliability of the role of the identified chloroplast-related genes on chloroplast development. It is speculated that the abnormal chloroplast development of the *chlm* mutant could be supervised and regulated by plant cells and thus induced the expression changes of chloroplast-related genes, trying to remedy the poor chloroplast state caused by the extreme impaired function of the *ChlM* gene.

### 2.9. Genes Regulating Hormone Regulatory Pathways Were Identified in the Chlm Mutant

Phytohormones were reported to perform important functions in regulating chloroplast biogenesis and development [55,56]. Due to the changes of hormone contents in the *chlm* leaves, expression alterations of hormone-related genes were investigated accordingly.

**Figure 8:**
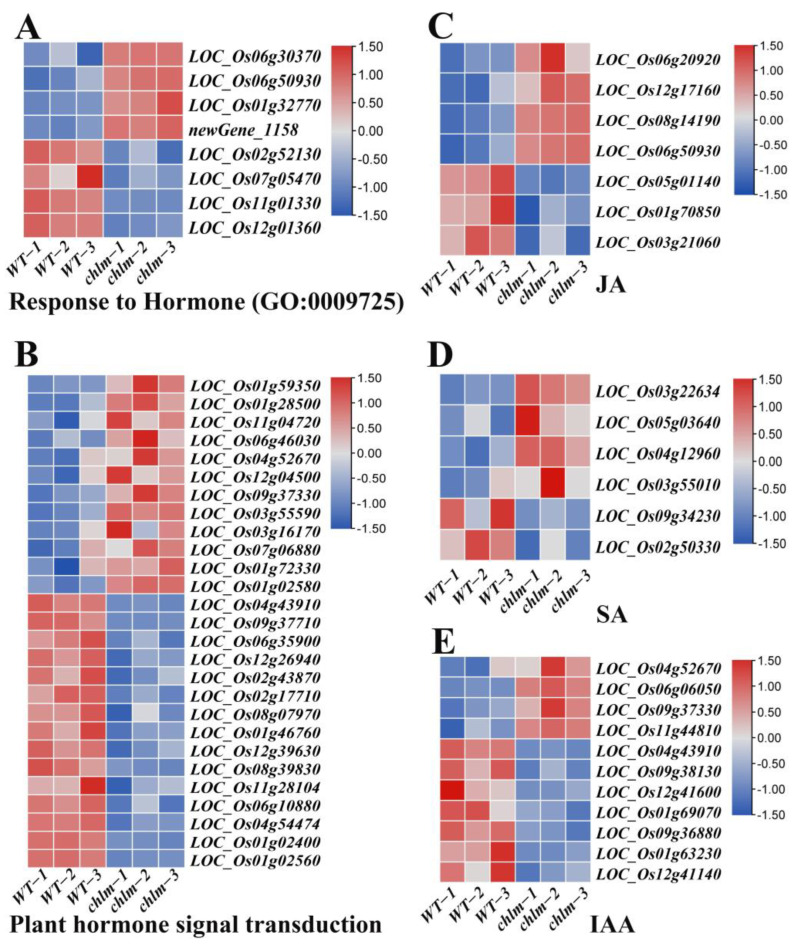
Expression patterns of DEGs involved in hormone pathways in the *chlm* mutant. (**A**) DEGs involved in response to hormone. (**B**) DEGs involved in plant hormone signal transduction pathways. (**C**) DEGs involved in JA metabolism or response. (**D**) DEGs involved in SA metabolism or response. (**E**) DEGs involved in IAA response or transport.

In this study, a total of 59 hormone-related DEGs were identified based on functional annotation, including 28 up-regulated genes and 31 down-regulated genes (Table S6). Of these 59 DEGs, eight genes were annotated to respond to hormone (GO:0009725) (Figure 8A, Table S6), and 27 genes were found to be involved in plant hormone signal transduction (ko04075) (Figure 8B, Table S6). Importantly, seven genes were annotated to be associated with JA metabolism or response, in which LOC_Os06g20920, LOC_Os12g17160, LOC_Os08g14190, LOC_Os05g01140, and LOC_Os01g70850 were involved in JA metabolism, while LOC_Os06g50930 and LOC_Os03g21060 were involved in JA response (Figure 8C, Table S6). Six genes were annotated to be associated with SA metabolism or response, in which LOC_Os03g22634, LOC_Os05g03640, LOC_Os04g12960, LOC_Os03g55010, and LOC_Os09g34230 were involved in SA metabolism, while LOC_Os02g50330 was involved in SA response (Figure 8D, Table S6). And 11 genes were annotated to be associated with IAA response or transport, in which LOC_Os04g52670, LOC_Os09g37330, LOC_Os11g44810, LOC_Os04g43910, LOC_Os12g41600, LOC_Os01g69070, and LOC_Os01g63230 were involved in IAA response, while LOC_Os06g06050, LOC_Os09g38130, LOC_Os09g36880, and LOC_Os12g41140 were involved in IAA transport (Figure 8E, Table S6). Among these genes, two genes LOC_Os06g20920 and LOC_Os05g01140 were identified and cloned as the *OsJMT* gene involving in JA metabolism (Table 2).

These results showed that the expression of hormone-related genes was regulated in the *chlm* mutant, implying that hormones might play important roles in pigment metabolism and chloroplast development regulated by the *ChlM* gene.

### 2.10. Genes Regulating Signal Pathways Were Identified in the Chlm Mutant

Signal transmission and transduction play an essential role in plant development [57]. It would be interesting to investigate the signal-related genes participating in the biological process regulated by the *ChlM* gene.

**Figure 9:**
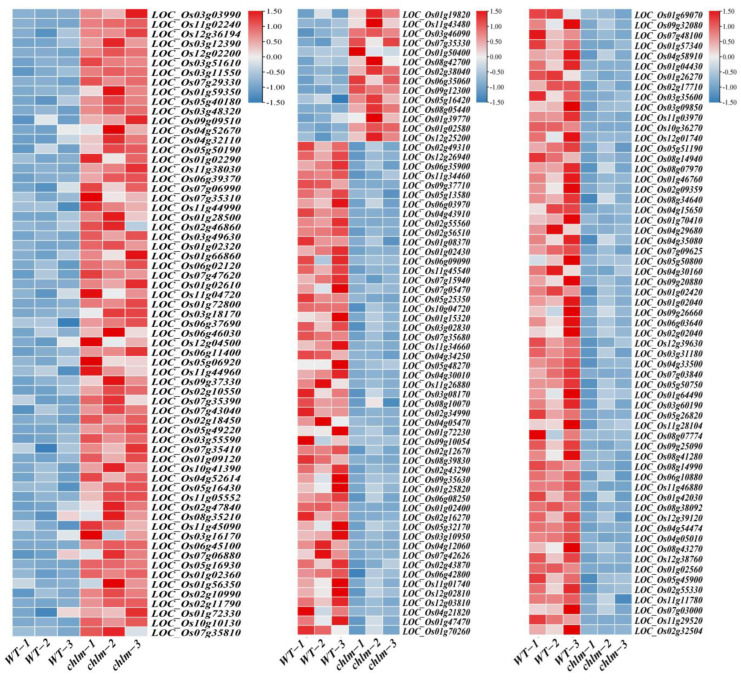
Expression patterns of DEGs involved in signal pathways in the *chlm* mutant. Genes referring to signal recognition or transduction are shown.

In this study, a total of 192 genes were annotated to be involved in signal recognition or transduction pathways, in which 79 genes displayed up-expression while 113 genes showed down-expression (Figure 9, Table S7). According to the search results from the China Rice Data Center, four signal-related genes including LOC_Os03g03990 (*OscpSRP43*), LOC_Os11g05552 (*YGL138(t)*), LOC_Os05g40180 (*OsSTN8*), and LOC_Os12g36194 (*WSL12*) were reported to play an essential role in chloroplast development and pigment accumulation (Table 2). These results showed that the expression of signal-related genes was regulated in the *chlm* mutant, implying that the signal pathway might play important roles in pigment metabolism and chloroplast development.

### 2.11. Transcription Factors Were Identified in the Chlm Mutant

Transcription factors are important regulators that activate or repress gene expression in a sequence-specific manner, playing an important role in gene expression regulation to various biological processes, including plant growth, development and stress responses [58,59]. It is speculated that transcription factors may be involved in regulating the gene expression of the *chlm* mutant.

**Figure 10:**
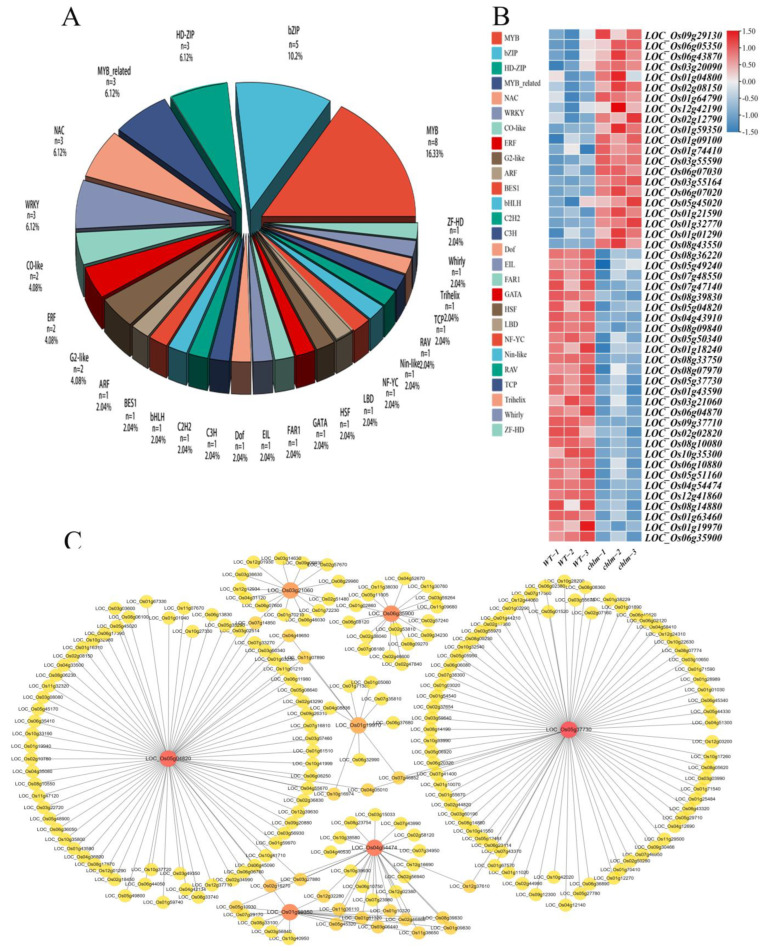
Identification of differentially expressed transcription factor genes in the *chlm* mutant. (**A**) Proportion of transcription factors. (**B**) Expression patterns of transcription factors in the wild type and *chlm* mutant. (**C**) The potential regulatory roles between seven transcription factors with identified DEGs.

Among the 1507 DEGs in this study, 49 DEGs were putatively identified as transcription factors associated with 27 families by searching the Plant Transcription Factor Database (http://planttfdb.cbi.pku.edu.cn/). The most abundant transcription factor family was the MYB superfamily (16.3%), which was followed by the bZIP family (10.2%), HD-ZIP family (6.1%), MYB-related family (6.1%), WRKY family (6.1%), and NAC family (6.1%) (Figure 10A). Furthermore, a total of 21 DEGs encoding transcription factors exhibited up-expression, while 28 transcription factors showed down-expression in the *chlm* mutant (Figure 10B, Table S8).

To further identify the potential regulatory roles between transcription factors with identified DEGs, all 1507 DEGs were investigated by searching the Plant Transcription Factor Database Regulatory Prediction Tool (http://planttfdb.cbi.pku.edu.cn/, accessed on 26 April 2023). Seven transcription factors (LOC_Os05g37730, LOC_Os05g04820, LOC_Os04g54474, LOC_Os06g35900, LOC_Os01g59350, LOC_Os01g19970, and LOC_Os03g21060) were found to be significantly associated with the tested DEGs (Figure 10C, Table S9). The MYB-type transcription factor LOC_Os05g37730 was predicted to regulate 76 DEGs, including six pigment metabolism-related genes, 19 chloroplast development-related genes, one hormone pathway-related gene, and six signal pathway-related genes (Figure 10C, Table S9). The MYB-type transcription factor LOC_Os05g04820 was predicted to regulate 73 DEGs, including one pigment metabolism-related gene, 10 chloroplast development-related genes, two hormone pathway-related genes, and seven signal pathway-related genes (Figure 10C, Table S9). The bZIP-type transcription factor LOC_Os04g54474 was predicted to regulate 26 DEGs, including one pigment metabolism-related gene, three chloroplast development-related genes, one hormone pathway-related gene, and two signal pathway-related genes (Figure 10C, Table S9). The bES1-type TF LOC_Os06g35900 was predicted to regulate 16 DEGs, including two chloroplast development-related genes, two hormone pathway-related genes, and three signal pathway-related genes (Figure 10C, Table S9). The bZIP-type TF LOC_Os01g59350 was predicted to regulate 16 DEGs, including one chloroplast development-related gene, one hormone pathway-related genes, and two signal pathway-related genes (Figure 10C, Table S9). The MYB-type TF LOC_Os01g19970 was predicted to regulate 10 DEGs, including three pigment metabolism-related genes and one signal pathway-related gene (Figure 10C, Table S9). The NAC-type TF LOC_Os03g21060 was predicted to regulate 14 DEGs, including three chloroplast development-related genes, one hormone pathway-related gene, and one signal pathway-related gene (Figure 10C, Table S9). These transcription factors might play important roles in chlorophyll synthesis and chloroplast development by regulating their target genes.

In addition, it is worth demonstrating that two transcription factors, LOC_Os06g05350 (*OsWHY1*, WHIRLY family) and LOC_Os02g12790 (*OsCGA1*, GATA family), were functionally validated and reported to be involved in chlorophyll synthesis and chloroplast development (Table 2). These results all imply that transcription regulation plays an important role in chlorophyll synthesis and chloroplast development regulated by the *ChlM* gene.

### 2.12. Verification by qRT-PCR of Some DEGs

**Figure 11:**
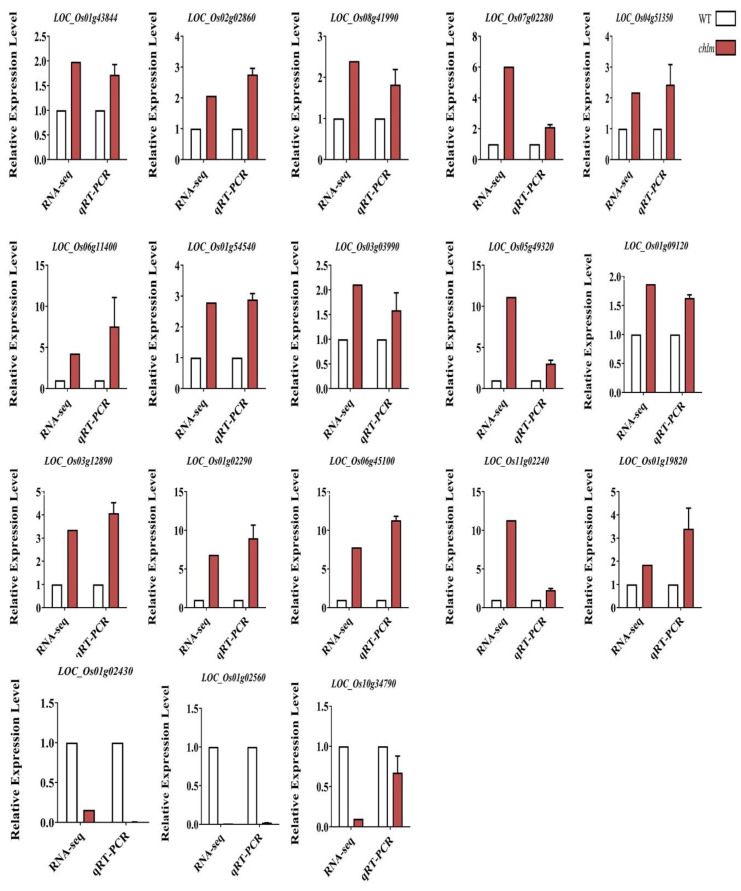
Validation of the RNA-Seq results by qRT-PCR. Eighteen DEGs were randomly selected for detection. Error bars indicate means ± SD of three biological replicates.

To identify the reliability of the transcriptomic data, 18 DEGs were randomly selected to detect their expression in the *chlm* leaves by qRT-PCR. The results showed that the expression patterns of all 18 genes detected by qRT-PCR were highly consistent with those from the Illumina sequencing data except for one gene LOC_Os10g34790 (Figure 11). These results demonstrated that the transcriptomic data are reliable.

## 3. Discussion

Chlorophyll content and chloroplast development are two critical factors for normal green leaf phenotype and photosynthesis. It is reported that gene mutation in the chlorophyll synthesis pathway usually leads to abnormal chloroplast development and eventually affects photosynthesis. It seems that these genes can also regulate the development of the chloroplast besides their roles in chlorophyll synthesis. However, the genetic regulatory network linking chlorophyll synthesis and chloroplast development is not very clear. In our previous study, map-based cloning of the *chlm* mutant identified the gene *ChlM* (LOC_Os06g04150) encoding the magnesium protoporphyrin IX methyltransferase (ChlM), which catalyzes the formation of MgPME from MgP and is a key speed-limiting enzyme in the chlorophyll synthesis pathway [18]. In this study, a physiological, cytological, and transcriptomic analysis of the *chlm* mutant was conducted to reveal the complex genetic regulatory network of the *ChlM* gene linking chlorophyll synthesis and chloroplast development.

### 3.1. Chlorophyll Metabolism Were Regulated by the ChlM Gene

Chlorophylls are the most abundant pigments used for harvesting energy from visible light in plants and then utilized for photosynthesis to support life forms on earth [60,61]. Due the lack of *ChlM* gene function, the *chlm* mutant leaves owned reduced chlorophyll content and thus showed a yellow leaf color (Figure 1A,B). This phenomenon is similar to other gene mutations in the chlorophyll synthesis pathway, such as *ChlD* [8], *ChlI* [8], *CRD1* [9], *ChlG* [11] and *OsCAO1* [13]. Chlorophyll metabolism is determined by complex biological processes, and genes in this process can be regulated in the *chlm* mutant. This study identified 62 chlorophyll-related DEGs in the *chlm* mutant leaves (Figure 6A–D). Glutamyl-tRNA is the initial substrate for the chlorophyll synthesis. *OsGluRS* was cloned to encode glutamyl-tRNA synthetase, and the mutation of this gene led to a chlorophyll deficiency phenotype [19]. LOC_Os02g02860 (*OsGluRS*) was found to be up-regulated in the *chlm* mutant (Table 2). Coproporphyrinogen III oxidase encoded by gene *RLIN1* could transform coproporphyrinogen III into protoporphyrinogen IX, which played an important role in the tetrapyrrole biosynthetic pathway [5]. In our study, LOC_Os04g52130 was identified to be gene *RLIN1*, which was up-expressed in the *chlm* mutant (Table 2). *OsChlD* encoded one of the subunits of the magnesium chelatase, which could convert protoporphyrin IX into Mg-protoporphyrin IX during chlorophyll synthesis [8]. Interestingly, LOC_Os03g59640 was identified to be *OsChlD* in this study (Table 2), and its expression level was up-regulated in the *chlm* mutant. Examining the motivation of the well-studied chlorophyll synthesis genes *OsGluRS*, *RLIN1*, and *OsChlD* in the *chlm* mutant implied that the functional decline of the ChlM protein could regulate the chlorophyll synthesis pathway itself to promote chlorophyll accumulation. Stroma-localized rice heme oxygenase 2 (OsHO2) is required for the oxidative cleavage of heme to biliverdin, which is the other branch of tetrapyrrole biosynthesis besides the chlorophyll biosynthesis branch [20]. And *OsHO2* (LOC_Os03g27770) was identified to be up-regulated in the *chlm* mutant (Table 2), which may be a feedback regulation pattern to reduce the accumulation of intermediate metabolites in chlorophyll biosynthesis branch due to the greatly weakened function of the chlm protein in the *chlm* mutant. *OsNYC3* encodes an alpha/beta fold hydrolase family protein, playing important roles in chlorophyll degradation [21]. *OsNYC3* (LOC_Os06g24730) was found to be down-regulated in the *chlm* leaves (Table 2), which might prevent the degradation of chlorophyll in this mutant.

In summary, the functional decline of the ChlM protein and decrease in chlorophyll content in the *chlm* mutant could cause the regulation of chlorophyll metabolism-related genes to compensate for the defect of chlorophyll synthesis in this mutant.

### 3.2. Chloroplast Development Was Regulated by the ChlM Gene

Chloroplasts are the place for photosynthesis, which usually consists of the chloroplast membrane, thylakoid and stroma in plants. The normal development of chloroplasts in higher plants requires the coordination of chloroplast genes and nuclear genes. Impaired function of the *ChlM* gene has caused the abnormal chloroplast development in the *chlm* mutant (Figure 1H–J). Whether the chlorophyll synthesis gene *ChlM* regulates chloroplast development-related genes is an interesting question.

Based on the transcriptomic data, a total of 278 chloroplast-related genes were identified to be regulated in the *chlm* mutant leaves (Figure 7). And interestingly, many of these genes were well studied and reported to be involved in chloroplast development. *WSP1* encoding a multiple organellar RNA editing factor protein is essential for chloroplast development by regulating plastid RNA editing and chloroplast ribosome biogenesis [22]. *OsTrxZ/wp2* encoding thioredoxin z regulates plastid RNA editing by interacting with multiple organellar RNA editing factors and contributes to chloroplast biogenesis in rice with an albino seedling lethality phenotype for the mutant [23,24]. *WLP2* encoding a plastid-encoded RNA polymerase-associated protein is required for chloroplast biogenesis under heat stress through interacting with the OsTrxZ protein in rice [25]. *OsPPR6* encoding a pentatricopeptide repeat protein for editing and splicing chloroplast RNA is required for chloroplast biogenesis in rice [26]. *CDE4* encodes a pentatricopeptide repeat protein involved in chloroplast RNA splicing and affects chloroplast development under low-temperature conditions in rice; and the gene mutant shows white leaf and defective chloroplast development at low temperature [27]. Two complementary recessive genes *etl1* and *etl2* encoding the homologous protein of HCF152 in *Arabidopsis* required for chloroplast RNA processing are found to control etiolation and chloroplast thylakoid development [28]. *WSL3* encoding a non-core subunit of plastid-encoded RNA polymerase is essential for early chloroplast development by interacting with subunits of the plastid-encoded RNA polymerase complex in rice [29]. *OsNUS1* encoding a plastid protein is involved in the regulation of chloroplast RNA metabolism and is essential for the build-up of the plastid genetic system during early chloroplast development under cold stress conditions [30]. The rice nuclear gene *WLP1* encoding a chloroplast ribosome L13 protein is needed for chloroplast development in rice grown under low-temperature conditions, and mutation of this gene leads to white leaf and abnormal chloroplast development [31]. Mutation of the rice *ASL2* gene encoding plastid ribosomal protein L21 caused chloroplast developmental defects and albino seedling death, suggesting the important role of this gene in chloroplast development [32]. *OsValRS2*, encoding a Val-tRNA synthetase for regulating chloroplast ribosome biogenesis in rice, is essential for early chloroplast development [33]. *ObgC* encoding a spo0B-related guanosine triphosphate binding protein that is crucial for chloroplast development and leaf greening plays a major role in the biosynthesis of plastid ribosomes during rice chloroplast development [34]. Nuclear encoded elongation factor *EF-Tu* is required for chloroplast development in rice grown under low-temperature conditions, and the gene mutant shows temperature-sensitive albino and disrupted chloroplast without thylakoid [35]. The nucleus-encoded chloroplast protein gene *YL1* is involved in chloroplast development and the efficient biogenesis of chloroplast ATP synthase in rice, and the mutation of this gene causes yellow leaf and abnormal chloroplast morphology [36]. *OscpSRP43* encoding a chloroplast signal recognition particle 43 protein is required for chloroplast development and photosynthesis, and the mutation of *OscpSRP43* induces a yellow–green leaf phenotype and impaired chloroplast development [37]. *YGL138(t)* encoding a putative signal recognition particle 54 kDa protein is involved in chloroplast development in rice [38]. *AL1* that encodes the sole octotricopeptide repeat protein plays an essential role in chloroplast development in rice, and the mutation of this gene results in albino leaves and a disrupted thylakoid structure [39]. *OsNOA1* functions in a temperature-dependent manner to regulate chlorophyll biosynthesis, rubisco formation and chloroplast development in rice, and the gene mutant has yellow leaves and vague thylakoid membranes [40]. The *VYL* gene encoding a plastid protein homologous to the *Arabidopsis* ClpP6 subunit plays an important role in the assembly of plastid caseinolytic protease and chloroplast development, and the gene mutant shows yellow leaves and a reduced thylakoid membrane [41]. *OsFd1* encoding the ferredoxin participates in photosynthetic electron transport, and the lost function of *OsFd1* leads to chloroplast degradation and leaf chlorosis [42]. The *OsFdC2* gene encoding a ferredoxin-like protein causes yellow leaves and an impaired chloroplast structure [43]. *OsSTN8* participates in the photosystem II repair mechanism and thus affects the chloroplast structure in rice [44]. *RNRS1* encoding the ribonucleotide reductase subunit for plastid DNA synthesis is necessary for chloroplast biogenesis during early leaf development [45]. *YLC1* encoding a DUF3353 superfamily protein is suggested to be involved in chlorophyll and lutein accumulation and chloroplast development at early leaf development in rice [46]. *WSL12* encoding a nucleoside diphosphate kinase 2 plays an important role in chloroplast development and chlorophyll synthesis in rice [47]. In this study, *WSP1* (LOC_Os04g51280), *OsTrxZ/wp2* (LOC_Os08g29110), *WLP2* (LOC_Os01g63220), *OsPPR6* (LOC_Os05g49920), *CDE4* (LOC_Os08g09270), *etl1* (LOC_Os11g01210), *etl2* (LOC_Os12g01210), *WSL3* (LOC_Os10g32540), *OsNUS1* (LOC_Os03g45400), *WLP1* (LOC_Os01g54540), *ASL2* (LOC_Os02g15900), *OsValRS2* (LOC_Os07g06940), *ObgC* (LOC_Os07g47300), *EF-Tu* (LOC_Os02g38210), *YL1* (LOC_Os02g05890), *OscpSRP43* (LOC_Os03g03990), *YGL138(t)* (LOC_Os11g05552), *AL1* (LOC_Os03g31150), *OsNOA1* (LOC_Os02g01440), *VYL* (LOC_Os03g29810), *OsFdC1* (LOC_Os03g45710), *OsFdC2* (LOC_Os03g48040), *OsSTN8* (LOC_Os05g40180), *RNRS1* (LOC_Os06g14620), *YLC1* (LOC_Os09g21250), and *WSL12* (LOC_Os12g36194) were all identified to be up-regulated in the *chlm* mutant (Table 2). So many well-studied genes were found in this study, which provided effective evidence for the roles of identified chloroplast-related DEGs on chloroplast development. It is speculated that the *chlm* mutant regulates the genes probably trying to compensate for chloroplast structure defects due to the impaired function of the ChlM protein in the mutant. It is proposed that chloroplast development was regulated by the *ChlM* gene in rice.

### 3.3. JA, SA and IAA May Play an Important Role in the Regulation Process of ChlM Gene to Chlorophyll Synthesis and Chloroplast Development

The biosynthesis of some plant hormones is highly related with chloroplasts. JA biosynthesis from linolenic acid begins in chloroplasts [62]. Plants synthesize SA through two pathways: the isochorismate acid synthase pathway and the phenylalanine ammonia lyase pathway, both of which originate from chloroplasts, use chorismic acid as a precursor, and refer to multiple enzyme catalysis [63]. Tryptophan dependent auxin synthesis is related to chloroplasts because tryptophan biosynthesis occurs in chloroplasts [64]. Plants synthesize ABA using the carotenoid pathway initiated from the cleavage of a C_40_ precursor known as β-carotene, and this process is mainly carried out in chloroplasts [65]. Accompanied by the changes of chloroplast structure, the increase in contents for JA, SA and IAA were observed in the *chlm* leaves, especially the significant increase for JA (Figure 2). And accordingly, 59 hormone-related DEGs were identified to be regulated in the *chlm* leaves (Figure 8, Table S6). DEGs related with JA, SA and IAA were mainly referring to the metabolism, response and transport of these hormones. However, the DEGs for the synthesis of JA, SA and IAA were not identified in the *chlm* mutant. The impaired function of the *ChlM* gene might not regulate these synthesis-related genes. The accumulation of these three hormones was possibly attributed to the increase in their substrate contents, which might be affected due to the impeded chlorophyll synthesis and chloroplast development of the *chlm* mutant. This speculation needs further measured experiments for related metabolites. The function of the accumulated JA, SA and IAA in the *chlm* mutant was an interesting question. *OsJMT* encoding the jasmonic acid carboxyl methyltransferase is involved in methylating JA to methyl jasmonate [48,49]. LOC_Os06g20920 and LOC_Os05g01140 were both identified as *OsJMT* genes in this study (Table 2). Methyl jasmonate and auxin were found to regulate the expression of chloroplast genes in barley [66]. Exogenous methyl jasmonate also played an important role in regulating the expression of chlorophyll synthesis genes to accumulate chlorophyll and improve the photosynthesis capacity in citrus [67]. And exogenous SA effectively improved the growth, photosynthesis, antioxidant enzyme activity, and stoma and chloroplast development in Dianthus *superbus* [68]. SA played protective roles in maintaining the integrity and function of photosynthetic photosystems in *Medicago sativa* [69]. It is reported that impaired chloroplast proteostasis (specifically for PSII proteins) may activate the chloroplast-established isochorismate pathway to produce SA; thus, SA is proposed to serve as a retrograde signaling molecule [70]. Considering the increases in JA, SA and IAA contents, and the expression changes of hormone-related genes, it is suggested that these three hormones may play an important role in the genetic network of the *ChlM* gene, regulating chlorophyll synthesis and chloroplast development.

### 3.4. Signal Pathways May Play an Important Role in the Regulation Process of ChlM Gene to Chlorophyll Synthesis and Chloroplast Development

The development and function of chloroplasts needs the involvement of enormous proteins encoded by the nucleus. And the status of chloroplasts is usually monitored by the nucleus. The processes that regulate the communication between nucleus and chloroplast, and the subsequent function execution of chloroplast, must need the involvement of massive genes related with signal pathways [71]. As expected, a large number of genes related with signal recognition and transduction were identified in the *chlm* mutant (Figure 9, Table S7). Some genes were cloned and verified to participate in chloroplast function. For example, rice chloroplast signal recognition particle 43 encoding gene *OscpSRP43* is required for chlorophyll synthesis, chloroplast development and photosynthesis [37]. In this study, LOC_Os03g03990 was identified to be *OscpSRP43* (Table 2). Similarly, a putative signal recognition particle 54 kDa protein-encoding gene *YGL138(t)* is involved in chlorophyll accumulation and chloroplast development [38]. And LOC_Os11g05552 was identified to be *YGL138(t)* in this study (Table 2). *OsSTN8* affects the phosphorylation of the core protein of photosystem II and is crucial for the repair of photosystem II in rice [44]. In the present study, an up-expressed gene LOC_Os05g40180 was identified as *OsSTN8* and was annotated to be involved in signal transduction mechanisms (Table 2, Table S7). The *WSL12* locus encoding the nucleoside diphosphate kinase OsNDPK2, with its mutation resulting in a white stripe leaf phenotype, plays an important role in chlorophyll biosynthesis and chloroplast development by regulating the expression of related genes [47]. In this study, an up-expressed gene LOC_Os12g36194 was identified to be gene *WSL12* and was annotated to be involved in the MAPK signaling pathway (Table 2, Table S7). It is suggested that signal pathway-related genes may play an important role in the regulation process of the *ChlM* gene to chlorophyll synthesis and chloroplast development.

### 3.5. Transcription Regulation May Play an Important Role in the Regulation Process of ChlM Gene to Chlorophyll Synthesis and Chloroplast Development

Transcription factors have been found to function on chlorophyll biosynthesis and chloroplast development in plants in a positive or negative manner [72,73]. In the *chlm* mutant, there were 49 transcription factors identified to show up- or down-expression (Figure 10, Table S8). Through predicting their target genes, seven transcription factors were found to target abundant DEGs, including those identified to be involved in pigment metabolism, chloroplast development, hormone regulation, and the signal pathways (Table S9). It is suggested that transcription factors can play an important role in the regulation process of the *ChlM* gene to chlorophyll synthesis and chloroplast development by regulating their target genes. In rice, some transcription factors were also cloned and found to be involved in chlorophyll synthesis and chloroplast development. *OsWHY1* encodes a WHIRLY family transcription factor protein, which interacts with OsTRXz to function on chloroplast RNA editing and splicing and eventually regulates chloroplast development by affecting the expression of chloroplast and ribosome development-related genes and chlorophyll synthesis-related genes [50]. In this study, LOC_Os06g05350 was identified as the *OsWHY1* gene, which was up-regulated in the *chlm* mutant (Table 2). *OsCGA1* encodes a GATA family transcription factor protein and is co-expressed with important nucleus-encoded chloroplast-localized genes, regulating chlorophyll accumulation and chloroplast biogenesis [51]. The ectopic expression of the *OsCGA1* gene in the rice bundle sheath could enhance its chloroplast biogenesis and increase the expression of photosynthesis-associated nuclear genes; and further activation of the endogenous *OsCGA1* gene by engineering its promoter could directly enhance chloroplast development [52]. In this study, LOC_Os02g12790 was identified as the *OsCGA1* gene, which was up-regulated in the *chlm* mutant (Table 2). These functionally validated transcription factor genes provided reliable evidence that transcription regulation played an important role in the regulation process of the *ChlM* gene, affecting chlorophyll synthesis and chloroplast development.

### 3.6. Possible Patterns for the Involvement of the ChlM Gene in the Retrograde Signaling Pathway

Plant cells coordinate their regulation of the expression of nuclear and plastid genes that encode components of the photosynthetic apparatus. The retrograde signaling from chloroplasts to the nucleus plays an important role in this coordinate control. It is proposed that MgP, the substrate of the *ChlM* gene, can act as a signaling molecule in the retrograde signaling pathway, and the accumulation of MgP is necessary to regulate the expression of many nuclear genes encoding photosynthesis-related chloroplastic proteins, including *LHCB1* [74]. However, a study re-evaluated this hypothesis by quantifying the correlation between the steady-state levels of MgP and MgPME with altered plastid signaling responses as monitored by the expression of *LHCB1*, *RBCS*, and *HEMA1* genes in a range of *Arabidopsis* mutants and conditions in which the steady-state levels of MgP and MgPME have been modified, and they found that there was no correlation between the steady-state levels of MgP (MgPME) with *LHCB1* expression or with any of the other genes tested [75]. Another study developed a sensitive liquid chromatography–mass spectrometry method to measure tetrapyrrole intermediates, and they also showed that there is no correlation between nuclear gene *LHCB1* expression with any of the chlorophyll biosynthetic intermediates including MgP over a range of growth conditions and treatments [76]. Consequently, these two studies negated the hypothesis that MgP acts as the retrograde signaling molecule to repress the expression of photosynthesis-related nuclear genes. Actually, in the rice *chlm* mutant, the expression level of a large number of pigment metabolism-related genes (Figure 6) and chloroplast protein-encoding genes (Figure 7) were regulated due to the impaired function of the *ChlM* gene, implying the fact that the retrograde signaling regulating this process absolutely exists. In this study, JA accumulated greatly in the *chlm* leaves (Figure 2A), and interestingly, the *chlm* mutant plants show an active growth state but not leaf senescence or death. Moreover, two *OsJMT* genes involved in methylating JA to form methyl jasmonate were also regulated in the *chlm* mutant (Table 2). Methyl jasmonate was found to function by regulating the expression of chloroplast genes in barley [66] and chlorophyll synthesis genes in citrus [67]. It is seemed that the accumulated JA or its downstream methyl jasmonate would play an important role in the regulatory process of chlorophyll synthesis and chloroplast development of the *chlm* mutant. Accordingly, we proposed that JA or its derivatives (such as methyl jasmonate) in a certain unknown state might act as a retrograde signaling molecule in one of the signaling pathways from the chloroplast to the nucleus. A previous report proposed that SA might serve as a retrograde signaling molecule in plants [70]. SA was found to accumulated in the *chlm* leaves (Figure 2B). And we also proposed that SA or its derivatives in a certain unknown state might act as a retrograde signaling molecule in one of the signaling pathways from chloroplasts to the nucleus. However, these speculations need further experiments for validation.

## 4. Materials and Methods

### 4.1. Plant Material and Sample Collection

The spontaneous mutant *chlm* in this study was derived from a photo-thermosensitive genic male sterile rice cultivar “guangzhan63S” (*Oryza sativa* L. subsp. *indica*) [18]. This mutant showed a yellow–green leaf phenotype and could be inherited stably. In our experiments, the seeds of wild-type and *chlm* mutant plants were soaked in a growth incubator at 37 °C until germinated. Then, the germinated seeds were grown in the experimental field with natural conditions. After 20 days of growth, the *chlm* mutant plants were easily distinguished by the yellow–green phenotype. For physiological, cytological, and RNA-seq experiments, the leaves of the wild-type and *chlm* mutant plants at the seedling stage were separately sampled. All the samples were collected from the uniform growth plants, flash frozen in liquid nitrogen and stored at −80 °C until use. Three biological replicates were performed in all experiments.

### 4.2. Determination of Chlorophyll and Hormone Contents

The chlorophyll content was determined according to the method as previously described [77]. In detail, the leaves sampled from the wild type and the *chlm* mutant were powdered in a pre-chilled mortar with liquid nitrogen. Then, approximately 0.1 g powder samples were mixed with 80% acetone to extract chlorophyll until the powder became white. During this process, the samples were kept in dark and periodic oscillation conditions. Next, the absorbance values of the extract were examined by a spectrophotometer at 663, 647 and 470 nm. Finally, the chlorophyll content was calculated by their corresponding formula.

Phytohormones including jasmonic acid (JA), salicylic acid (SA), indoleacetic acid (IAA), and abscisic acid (ABA) were extracted and measured following a previously described method [78]. In brief, the powder samples were mixed with Bieleski solvent (methanol/water) to extract hormones, after which we added the internal standards of these four hormones, and next, the extract solution was detected using a nano-LC-ESI-Q-TOF-MS system to determine hormones.

### 4.3. Transmission Electron Microscopic Observation

The leaves dissected from the wild-type and the *chlm* mutant were cut into smaller segments. Then, the leaf segments were fixed with 2.5% glutaraldehyde in sodium phosphate buffer (pH 7.2) for 4 h at 4 °C and postfixed with 1% (*v*/*v*) osmium tetroxide in PBS for an additional 2 h. Then, the tissues were dehydrated through an acetone series and embedded in Spurr’s resin according to the previously described method [46]. Subsequently, for ultrastructural observations of leaf chloroplasts, 70 nm thick sections were cut with a Leica EM UC6 ultramicrotome and stained with 1% (*w*/*v*) uranyl acetate and 1% (*w*/*v*) lead citrate. At last, leaf cells were observed and photographed using a transmission electron microscope.

### 4.4. RNA Extraction and Preparation of cDNA Library

The total RNA was extracted from leaves of the wild type and the *chlm* mutant using TRIZOL reagent following the manufacturer’s instructions. Three biological replicates were analyzed for each RNA sample. RNA degradation and contamination were monitored on 1% agarose gel electrophoresis. RNA purity was tested by a NanoPhotometer spectrophotometer (IMPLEN, Munich, Germany). RNA concentration and integrity were measured using a Qubit RNA Assay Kit in a Qubit 2.0 Flurometer (Life Technologies, Carlsbad, CA, USA) and Agilent Bioanalyzer 2100 system (Agilent Technologies, Santa Clara, CA, USA). RNA concentration and purity were measured using a NanoDrop 2000 (Thermo Fisher Scientific, Waltham, MA, USA). RNA integrity was assessed using the RNA Nano 6000 Assay Kit of the Agilent Bioanalyzer 2100 system (Agilent Technologies, Santa Clara, CA, USA).

After qualification of the RNA quality test, the libraries were constructed following the procedures. A 1 µg RNA sample was used as the input material for library preparation. Sequencing libraries were generated using an NEBNext Ultra^TM^ RNA Library Prep Kit for Illumina (NEB, Ipswich, MA, USA) following the manufacturer’s recommendations, and index codes were added to attribute sequences to each sample. In detail, mRNA was purified using poly (dT) oligo-attached magnetic beads. Afterwards, mRNA was randomly broken into fragments. Then, the first-strand cDNA was synthesized using a random hexamer primer and M-MLV reverse transcriptase. Second-strand cDNA synthesis was subsequently performed using DNA polymerase I and RNase H. Next, the double-strand cDNA was subjected to end-repair, dA tailing and adaptor ligation. Finally, suitable fragments were isolated and then enriched using polymerase chain reaction amplification. Furthermore, the quality of obtained libraries was assessed on the Agilent Bioanalyzer 2100 system.

### 4.5. Illumina Deep Sequencing and Data Analysis

After the libraries were qualified, the clustering of the index-coded samples was performed on a cBot cluster generation system using a TruSeq PE Cluster Kit v4-cBot-HS (Illumina, San Diego, CA, USA) according to the manufacturer’s instructions. After cluster generation, the constructed libraries were sequenced on an Illumina Hiseq 2500 platform, and paired-end reads were generated. Library construction and sequencing were performed by Beijing Biomarker (BMKCloud) Technology Co., Ltd. (Beijing, China). Finally, the raw reads were generated.

To obtain the clean reads, the adaptor sequences, reads containing ploy-N and low-quality reads were removed from raw reads using Perl script. Simultaneously, the GC content, Q20 and Q30 of the clean reads were calculated. All the clean reads with high quality were used for the identification of mRNAs. Subsequently, the clean reads were mapped to the rice reference genome (http://rice.plantbiology.msu.edu/, accessed on 1 August 2022) using TopHat2 V 2.1.0 software [79]. The read counts for each gene were calculated with Cufflinks V2.0 software, and gene expression levels were estimated as Fragments Per Kilobase of transcript per Million fragments mapped (FPKM) values.

### 4.6. Identification and Functional Enrichment Analysis of Differentially Expressed Genes

Prior to identifying the interested and reliable differentially expressed genes (DEGs), three biological replicates were performed. The correlations of samples were evaluated by Pearson’s correlation coefficient [80]. Furthermore, DESeq R package [81] was performed to identify DEGs between the wild type and the *chlm* mutant. DESeq provides statistical routines for identifying differential expression in gene expression data using a model based on the negative binomial distribution. To control for the false discovery rate (FDR), *p*-values obtained from DESeq were adjusted using the edgeR approach. Genes with the fold change ≥ 2 and FDR < 0.01 were considered to be differentially expressed.

To explore the potential functions of DEGs, the functional enrichment analysis was performed. Gene Ontology (GO) enrichment analysis was performed using the GOseq R package [82]. And GO terms with *p*-values < 0.05 were considered significantly enriched. To further investigate the involvement in pathways of DEGs, Kyoto Encyclopedia of Genes and Genomes (KEGG) enrichment analysis was performed using KOBAS V3.0 software. And the pathways with the *p*-value < 0.05 were considered to be statistically significantly enriched. In addition, the heatmap analysis of DEGs was carried out using TBtools V2.012 software. The FPKM values of DEGs were imported into TBtools, and the data were calculated by Log2 and then clustered to obtain the resulting figures. Furthermore, protein–protein interaction analysis of DEGs was performed, and the result was visualized through Cytoscape V3.9.1 software.

### 4.7. Quantitative Real-Time PCR (qRT-PCR) Analysis

To validate the expression patterns of DEGs, quantitative real-time PCR (qRT-PCR) was performed. The total RNA of rice leaf samples was extracted using a TRIZOL reagent (Invitrogen, Waltham, MA, USA) according to the manufacturer’s instructions. After extraction, the total RNA was subjected to reverse transcription using a PrimeScript™ RT reagent Kit with gDNA Eraser (Takara, Beijing, China) according to manufacturer’s instructions. Then, qRT-PCR was performed using a SYBR Green PCR kit (Takara, Beijing, China) on a CFX96 Real-Time PCR system (Bio-Rad, Hercules, CA, USA) to validate the expression levels of DEGs. Three genes, UBC (LOC_Os02g42314), ARF (LOC_Os05g41060) and Profilin-2 (LOC_Os06g05880) were used as internal reference genes to normalize the qRT-PCR data [83]. Furthermore, qRT-PCR conditions were as follows: 10 min at 95 °C for pre-denaturation followed by 40 cycles of 10 s at 95 °C, 10 s at 60 °C, and 15 s at 72 °C and then a melt curve from 65 to 95 °C. The relative RNA expression levels were calculated by using the CFX Manager 3.1 (Bio-Rad) with the 2^−ΔΔCT^ method. The reaction was carried out using three biological replicates with three technical replicates. All primers are shown in Table S10.

## 5. Conclusions

In this study, we analyzed changes in pigment contents, chloroplast structure and hormone contents in the *chlm* mutant and then used transcriptomic data to reveal the *ChlM* gene involved regulatory network linking chlorophyll synthesis with chloroplast development. Genes related to pigment metabolism, chloroplast development, hormone pathways, signal pathways and transcriptional regulation were found to function in this regulation process, revealing a complex genetic regulatory network linking chlorophyll synthesis and chloroplast development. The *ChlM* gene involved in retrograde signaling was discussed. Jasmonic acid, salicylic acid or their derivatives in a certain unknown state were proposed as retrograde signaling molecules in one of the signaling pathways from the chloroplast to the nucleus.

## Figures and Tables

**Table 1:** Characteristics of RNA-sequencing data in all six samples.

Samples	WT-1	WT-2	WT-3	*chlm*-1	*chlm*-2	*chlm*-3
Total reads	56,640,244	44,565,826	43,136,908	44,053,700	40,418,298	40,776,152
Clean reads	28,320,122	22,282,913	21,568,454	22,026,850	20,209,149	20,388,076
Clean bases	8,411,102,720	6,636,988,544	6,420,743,624	6,551,792,242	6,010,508,590	6,071,105,796
Q20 (%)	93.3	93.29	93.22	93.22	93.3	93.24
Q30 (%)	85.18	85.11	85.06	85.07	85.12	85.06
GC (%)	54.56	54.59	54.85	55.14	54.67	54.74
Total mapped	86.94%	86.94%	86.91%	86.72%	86.94%	87.15%
Uniquely mapped	83.56%	83.42%	83.93%	82.61%	83.68%	83.49%
Multiple mapped	3.38%	3.52%	2.98%	4.10%	3.26%	3.66%

**Table 2:** The well-studied functionally validated genes between WT and the *chlm* mutant.

MSU-ID	Gene Name	Description	Expression Patterns	Reference
Chlorophyll metabolism				
LOC_Os02g02860	*OsGluRS*; *Cde1(t)*	Glutamyl tRNA synthetase	up	[19]
LOC_Os04g52130	*RLIN1*; *LLM1*	Coproporphyrinogen III oxidase	up	[5]
LOC_Os03g59640	*OsChlD*; *ygl3*	Magnesium chelatase D subunit	up	[8]
LOC_Os03g27770	*OsHO2*; *OsYLC2*	Heme oxygenase	up	[20]
LOC_Os06g24730	*OsNYC3*	Alpha/beta fold hydrolase family protein	down	[21]
Chloroplast development				
LOC_Os04g51280	*WSP1*; *OsMORF2*	Multiple organellar RNA editing factor	up	[22]
LOC_Os08g29110	*OsTrxZ*; *wp2*	Subunit of PEP (plastid-encoded RNA polymerase) in chloroplasts	up	[23,24]
LOC_Os01g63220	*WLP2*; *OsFLN1*	Plastid-encoded RNA polymerase associated protein	up	[25]
LOC_Os05g49920	*OsPPR6*	Triangular pentapeptide repeat protein	up	[26]
LOC_Os08g09270	*CDE4*	A pentapeptide repeat protein	up	[27]
LOC_Os11g01210	*etl1*	Etiolation gene	up	[28]
LOC_Os12g01210	*etl2*	Etiolation gene	up	[28]
LOC_Os10g32540	*WSL3*	Non-core subunit of plastid-encoded RNA polymerase	up	[29]
LOC_Os03g45400	*OsNUS1*	Plastid protein	up	[30]
LOC_Os01g54540	*WLP1*	A chloroplast ribosome L13 protein	up	[31]
LOC_Os02g15900	*ASL2*	A plastid 50S ribosomal protein L21	up	[32]
LOC_Os07g06940	*OsValRS2*	Val-tRNA synthetase	up	[33]
LOC_Os07g47300	*ObgC*	A spo0B-associated GTP binding protein	up	[34]
LOC_Os02g38210	*EF-Tu*	Translation elongation factor	up	[35]
LOC_Os02g05890	*YL1*	A nucleus encoded chloroplast protein	up	[36]
LOC_Os03g03990	*OscpSRP43*	A chloroplast signal recognition particle 43	up	[37]
LOC_Os11g05552	*YGL138(t)*	Putative signal recognition particle 54 kDa protein	up	[38]
LOC_Os03g31150	*AL1*	The sole octotricopeptide repeat protein	up	[39]
LOC_Os02g01440	*OsNOA1*	Nitric oxide synthesis-related protein	up	[40]
LOC_Os03g29810	*VYL*	A chloroplast protein	up	[41]
LOC_Os03g45710	*OsFd1*	Ferredoxin	up	[42]
LOC_Os03g48040	*OsFdC2*	Ferredoxin-like protein	up	[43]
LOC_Os05g40180	*OsSTN8*	Serine/threonine protein kinase	up	[44]
LOC_Os06g14620	*RNRS1*	A small subunit of ribonucleotide reductase	up	[45]
LOC_Os09g21250	*YLC1*	A DUF3353 superfamily gene	up	[46]
LOC_Os12g36194	*WSL12*	Nucleoside diphosphate kinase 2	up	[47]
Hormone regulatory pathways				
LOC_Os06g20920	*OsJMT*	Jasmonic acid carboxyl methyltransferase gene	up	[48]
LOC_Os05g01140	*OsJMT*	Jasmonic acid carboxyl methyltransferase	down	[49]
Signal pathway				
LOC_Os03g03990	*OscpSRP43*	A chloroplast signal recognition particle 43	up	[37]
LOC_Os11g05552	*YGL138(t)*	Putative signal recognition particle 54 kDa protein	up	[38]
LOC_Os05g40180	*OsSTN8*	Serine/threonine protein kinase	up	[44]
LOC_Os12g36194	*WSL12*	Nucleoside diphosphate kinase 2	up	[47]
Transcription factor				
LOC_Os06g05350	*OsWHY1*	WHIRLY family transcription factor	up	[50]
LOC_Os02g12790	*OsCGA1*	GATA family transcription factor	up	[51,52]

## Data Availability

Data described in the manuscript are available from the corresponding author on reasonable request.

## Supplementary Materials

The following supporting information can be downloaded at https://www.mdpi.com/article/10.3390/plants12213785/s1, Table S1. The detailed information of 1534 DEGs between wild type and *chlm* mutant; Table S2. GO and KEGG enrichment analysis of 1534 DEGs; Table S3. The detailed information of the top 40 PPI protein-encoding genes; Table S4. The detailed information of genes related to pigment metabolism; Table S5. The detailed information of genes related to chloroplast development; Table S6. The detailed information of genes related to hormone pathways; Table S7. The detailed information of genes related to signal pathways; Table S8. The detailed information of genes related to transcription factors; Table S9. The potential regulatory roles between transcription factors with DEGs; Table S10. All primers used for qRT-PCR analysis.

## Abbreviations

ChlM	Magnesium protoporphyrin IX methyltransferase
MgP	Magnesium protoporphyrin IX
MgPME	Magnesium protoporphyrin IX methyl ester
WT	Wild type
JA	Jasmonic acid
SA	Salicylic acid
IAA	Indole acetic acid
ABA	Abscisic acid
DEGs	Differentially expressed genes
FPKM	Fragments Per Kilobase of transcript per Million fragments mapped
FDR	False discovery rate
GO	Gene Ontology
KEGG	Kyoto Encyclopedia of Genes and Genomes
PPI	Protein–protein interaction
qRT-PCR	Quantitative real-time PCR
